# AGING REPROGRAMMED

**DOI:** 10.1093/geroni/igae098.0867

**Published:** 2024-12-31

**Authors:** Heinrich Jasper

**Affiliations:** Chair

## Abstract

Epigenetic reprogramming of differentiated cells into stem-like or plastic cells is a feature of regenerative processes in many tissues. This process can be leveraged to induce full pluripotency, which essentially represents a fully rejuvenated state of the epigenome. Evidence is now accumulating that partial in vivo epigenetic reprogramming, where reprogramming to pluripotency is initiated but not completed, can alleviate age-related dysfunction in tissue repair, and even extend lifespan. In a wide range of disease models, reprogramming has been shown to improve outcomes and promote tissue repair. This symposium will explore fundamental cellular mechanisms of partial reprogramming, the tissue consequences of in vivo reprogramming, and discuss the potential therapeutic impact of reprogramming on age-related diseases.

